# TiO_2_ Modification with Transition Metallic Species (Cr, Co, Ni, and Cu) for Photocatalytic Abatement of Acetic Acid in Liquid Phase and Propene in Gas Phase

**DOI:** 10.3390/ma12010040

**Published:** 2018-12-23

**Authors:** Ana Amorós-Pérez, Laura Cano-Casanova, Ana Castillo-Deltell, María Ángeles Lillo-Ródenas, María del Carmen Román-Martínez

**Affiliations:** MCMA Group, Department of Inorganic Chemistry and Materials Institute, University of Alicante, E-03080 Alicante, Spain; ana.amoros@ua.es (A.A.-P.); laura.cano@ua.es (L.C.-C.); ana.castillo@hotmail.es (A.C.-D.); mcroman@ua.es (M.d.C.R.-M.)

**Keywords:** photocatalysis, TiO_2_, P25, transition metals, acetic acid, propene.

## Abstract

The commercial P25 titania has been modified with transition metallic species (Cr, Co, Ni, and Cu), added by impregnation with aqueous solutions of the corresponding nitrates. The preparation procedure also includes a heat treatment (500 °C) in argon to decompose the nitrates, remove impurities and to strengthen the metal–TiO_2_ interaction. The catalysts have been thoroughly characterized using N_2_ adsorption, scanning electron microscopy (SEM), X-ray diffraction (XRD), UV-visible diffuse-reflectance spectroscopy (UV-vis DRS) and X-ray photoelectron spectroscopy (XPS), and have been tested in the aqueous phase decomposition of acetic acid and in the gas phase oxidation of propene, using an irradiation source of 365 nm in both cases. The photocatalytic activity of the four metal-containing catalysts varies with the nature of the metallic species and follows a similar trend in the two tested reactions. The effect of the nature of the added metallic species is mainly based on the electrochemical properties of the supported species, being Cu/P25 (the sample that contains copper) the best performing catalyst. In the photodecomposition of acetic acid, all the metal-containing samples are more active than bare P25, while in the gas phase oxidation of propene, bare P25 is more active. This has been explained considering that the rate-determining steps are different in gas and liquid media.

## 1. Introduction

With the increasing concern in the use of renewable resources and in finding better ways to use solar energy, solar light-activated photocatalysis has become an attractive tool. In particular, heterogeneous photocatalysis is a process of great potential for pollutants abatement in gas and liquid phases. This method has considerable advantages over some existing technologies: it destroys pollutants rather than transferring them to another phase; it usually leads to complete mineralization of organic pollutants into CO_2_, H_2_O and innocuous mineral salts; it operates at ambient conditions, with any type of substrate, without complex processing requirements and it can be easily implemented in both aqueous and gaseous applications [1,2].

In this work, heterogeneous photocatalysis, a promising advanced oxidation technology for removing contaminants at trace levels [3], is selected to address the problem of two reactions of environmental interest: acetic acid decomposition and propene oxidation. In wastewater, several bacteria produce acetic acid (HAc) as a primary fermentation product [4]. However, due to its high chemical stability and/or low biodegradability [5], HAc is a recalcitrant compound quite difficult to decompose using conventional methods. Catalytic photodegradation is an interesting technique used for the degradation of recalcitrant contaminants, such as acetic acid, because of its relatively low cost and because it can be performed in mild operation conditions [6]. Furthermore, the photodecomposition of HAc generates gaseous products of energetic interest (mainly CH_4_ and CO_2_, and H_2_ in minor concentration) [7]. In this way, the transformation of water pollutants into syngas components and/or fuel compounds would be desirable from both the ecological and economical points of view. On the other hand, propene is a common air pollutant belonging to the category of volatile organic compounds (VOCs) that is involved in photochemical smog [8] and is one of the major sources of indoor air pollution, as it is an important component of tobacco smoke [9]. Hence, its photo-oxidation is very interesting from an environmental point of view. However, the photocatalytic conversion of the two mentioned substrates is still far from practical implementation because of the lack of efficient and cheap catalysts [10]. 

TiO_2_ has emerged as one of the most promising semiconductor photocatalysts, as it is stable also in aqueous media, tolerant to both acidic and alkaline solutions, it is recyclable and relatively simple to prepare, and shows good ability for air and water purification [11]. However, a limitation of TiO_2_ is that the photogenerated e^−^/h^+^ pairs undergo a rapid recombination, therefore decreasing the photocatalytic efficiency of the system. Besides, TiO_2_ has a relatively wide band gap (3.0 eV for rutile and 3.2 eV for anatase phase [12]), that lies in the UV radiation region and, as a result, only 5% of sunlight photons can be harvested to activate TiO_2_ [13].

The addition of transition metallic species to TiO_2_ catalysts has shown to be useful to reduce the photogenerated e^−^/h^+^ recombination rate and, also, to improve the photoresponse to the visible light [14,15,16]. The band gap and electrochemical properties of TiO_2_ are known to be modified when transition metallic ions replace Ti (IV) centers (substitutional doping), occupy interstitial sites (interstitial doping) or form aggregates on the TiO_2_ surface [17]. Substitutional doping can occur in titanium dioxide when the difference between the atomic radii of the dopant and of Ti (IV) is less than 15% [18]. The introduction of dopants in the TiO_2_ lattice causes local distortion of the crystal structure, thus altering the crystallinity degree and phase transformation. Doping (substitutional or interstitial) can also generate trap levels, which modify the band gap and allow the capture of the photo-induced e^−^ in the conduction band (CB) of TiO_2_, leaving the h^+^ in the valence band (VB) of TiO_2_. This hinders the e^−^/h^+^ pairs recombination and, thus, promotes the photocatalytic efficiency [14,16,19,20]. Additionally, metallic aggregates not chemically bonded to TiO_2_ can act as electrons scavengers, also preventing the recombination of h^+^/e^−^ pairs. On the other hand, when a metal with a certain work function (energy required for moving an electron from a Fermi level to the local vacuum level [21]) is put in contact with a semiconductor of lower work function value, it provides a Schottky barrier that facilitates the transfer of electrons from the semiconductor to the metal. Thus, the metal serves as an electron trap which prevents electron migration to the semiconductor, avoiding recombination [22]. The ability of metal ions to act as effective traps is related, among others, with their electrochemical properties, the metal concentration and the intensity of the incident light [22,23,24]. Considering all this, and with the purpose of enhancing the activity, the added transition metal ions should have a work function higher than that of TiO_2_.

Although noble metals (such as Pt, Pd or Au) have proved to be effective to enhance the photocatalytic activity of TiO_2_ [25], they are unsuitable for large-scale commercial use due to their limited availability and high cost. In contrast, earth-abundant non-noble transition metal-based materials are promising alternatives to enhance the TiO_2_ performance. For example, transition metals, such as Cu, Co, Ni, Cr, Fe, Mn and V [14,16,26] have been reported to decrease the TiO_2_ band gap and to extend its photo-response to the visible region.

The use of non-noble transition metals/TiO_2_ systems for the photocatalytic degradation of organic pollutants in the liquid and gas phase has been described by several authors [14,16,19,20,27]. The incorporation of metallic species to titania has been often performed during the TiO_2_ synthesis [14,16,26,28], and relatively few studies report the incorporation of metallic species by impregnation over previously synthesized TiO_2_ [19,27]. However, the conclusions about the role of the incorporated metal are sometimes controversial. For example, Di Paola, et al. [27] studied the 4-nitrophenol photodegradation in aqueous suspension and found a decrease of the TiO_2_ photoactivity when it contains Co, Cr, Cu, Fe, Mo and V ions added by impregnation. In contrast, Tayade et al. [19] found that the photocatalytic activity of Fe, Ni and Ag containing TiO_2_ in the degradation of acetophenone and nitrobenzene in aqueous solution was higher than that of bare TiO_2_, while the presence of Co or Cu led to a lower photoactivity.

All these considerations prompted us to prepare a series of M/TiO_2_ photocatalysts (M = Cr, Co, Ni and Cu) by impregnation of the inexpensive, versatile and commercial P25 titania (from Degussa). These new materials were characterized by N_2_ adsorption isotherms, scanning electron microscopy (SEM), X-ray diffraction (XRD), UV-visible diffuse-reflectance spectroscopy (UV-vis DRS) and X-ray photoelectron spectroscopy (XPS) analysis, and their photocatalytic activity was investigated in the photo-degradation of aqueous acetic acid into CO_2_, CH_4_ and H_2_ and in the gas phase photo-oxidation of propene. The overall performances were discussed in terms of activity and selectivity as a function of the properties of the photocatalysts. 

## 2. Materials and Methods

### 2.1. Preparation of M/P25 Samples

Degussa Aeroxide P25 TiO_2_ has been used as a raw material because of its good activity in many photocatalytic reactions. Cr, Co, Ni, and Cu were incorporated to P25 by impregnation as follows: P25 (2 g) was put in contact with an aqueous solution (5 mL) of Cr(NO_3_)_3_·9H_2_O, Co(NO_3_)_2_·6H_2_O, Ni(NO_3_)_2_·6H_2_O or Cu(NO_3_)_2_·3H_2_O of the appropriate concentration to obtain 1 wt.% metal loading. The mixture was stirred (2 h) and sonicated (30 min) and, then, the solvent excess was removed (80 °C, 24 h).

All the samples were heat-treated in argon atmosphere (90 mL/min, 5 °C/min, 500 °C, 2 h) to decompose the nitrates, remove impurities and to strengthen the metal–TiO_2_ interaction. The Ar atmosphere was selected according to the previous work [29]. The used nomenclature is M/P25-Ar (M = Cr, Co, Ni or Cu). P25 was wetted and heat treated in the same conditions as M/P25-Ar samples to use it as reference; this sample is named P25-Ar. 

### 2.2. Characterization

Porosity (textural properties) of the photocatalysts has been charaterized by physical adsorption of gases. N_2_ adsorption–desorption at −196 °C (after degasification (250 °C, 4 h)) was measured in a Quantachrome Autosorb-6B equipment (Quantachrome Instruments, Boynton Beach, FL, USA). The specific BET surface area (S_BET_) and the micropore volume (V_N2_) were determined by applying, respectively, the Brunauer–Emmett–Teller (BET) or Dubinin–Radushkevich equations to the N_2_ adsorption isotherms. These determinations have an associated error of 2%. Mesopore volume (V_meso_) was estimated by the difference in the volume of N_2_ adsorbed as liquid at P/P_0_ = 0.9 and P/P_0_ = 0.2. The total pore volume (V_T_) was determined by the nitrogen adsorption volume at a P/P_0_ (relative pressure) of 0.99. For such calculation, the density of liquid nitrogen at −196 °C was used. The average pore size (Ø) was calculated from the nitrogen adsorption isotherms by the Barret-Joyner-Halenda (BJH) method [30,31,32].

SEM characterization was performed using the JSM-840 JEOL equipment (JEOL Ltd., Tokyo, Japan) and the scanning electron microscopy with energy dispersive X-ray (SEM-EDX) mapping was conducted with a Hitachi S3000N microscope (Hitachi, Tokyo, Japan) and the Bruker XFlash 3001 X-ray energy dispersive spectrometer (Bruker, Billerica, MA, USA). 

XRD analysis using Cu Kα (1.54 Å) radiation was performed in a SEIFERT 2002 equipment (Rich.Seifert & Co., Ahrensburg, Germany), in the angular 2θ range 6–80° and with a scanning velocity of 2°/min. The experimental errors are within ± 0.1°. The average crystallite size, referred to as crystal size, was calculated by Scherrer’s equation (Equation (1)) [33]:

(1) $$B=\frac{K\lambda }{\beta cos\theta }$$

where B is the average crystallite size (nm); K is a constant, with a chosen value of 0.9; λ is the wavelength of the radiation source; β is the full width at half maximum intensity (FWHM) and θ is the Bragg angle of the maximum intensity peak. The amount of crystalline anatase (A_Cryst_) and rutile (R_Cryst_) has been calculated using the equations proposed by Jensen et al. [34]:

(2) $$A_{\mathrm{Cryst}}=\frac{\frac{A_{\mathrm{Anatase}\left(101\right)}}{A_{{\mathrm{CaF}}_{2}\left(220\right)}}\times 100}{1.25}$$

(3) $$R_{\mathrm{Cryst}}=\frac{\frac{A_{\mathrm{Rutile}\left(110\right)}}{A_{{\mathrm{CaF}}_{2}\left(220\right)}}\times 100}{0.90}$$

where A_Anatase (101)_, A_Rutile(110)_ and A_CaF2(220)_ are the peak areas determined in XRD spectra measured for a 50/50 (w/w) TiO_2_/CaF_2_ mixture, and 1.25 and 0.90 are the ratios between areas of the same peaks when a 100% anatase or 100% rutile crystalline TiO_2_ sample (respectively) is used. The percentage of crystalline (W_Cryst_) and amorphous (W_Am_) phases present in the photocatalysts were determined as indicated in Equations (4) and (5), respectively.

(4) $$W_{\mathrm{Cryst}}=A_{\mathrm{Cryst}}+R_{\mathrm{Cryst}}$$

(5) $$W_{\mathrm{Am}}=100-W_{\mathrm{Cryst}}$$

Diffuse reflectance spectra (DRS) were determined by means of a Jasco V-670 UV-vis spectrophotometer (JASCO, Inc., Pfungstadt, Germany), using BaSO_4_ as the reference sample. The spectra were recorded at room temperature in air, in the 200–800 nm range. The absorption edge wavelength was estimated from the intercept at zero absorbance of the high slope portion of each spectrum in the 200–800 nm range (absorbance method). The band gap energy was calculated using three different methods: absorbance, indirect and direct ones. In the absorbance method, the band gap can be calculated using Equation (6), where E_g_ is the band gap energy (eV) and *λ* is the edge wavelength (nm) [35].

(6) $$E_{g}=\frac{1239.8}{\lambda }$$

In the direct and indirect methods, the band gap values are obtained from the Kubelka-Munk function of the reflectance, plotting, respectively, (F(R)hυ)^2^ vs. hυ or (F(R)hυ)^0.5^ vs. hυ [36].

XPS spectra were obtained using a K-Alpha spectrophotometer (Thermo-Scientific, Waltham, MA, USA) with a high-resolution monochromator and the following specifications: Al anode (1486.6 eV) X-ray source, 5 × 10^−9^ mbar analysis chamber pressure and detection in constant energy mode with pass energy of 200 eV for the survey spectrum, and of 50 eV for the sweep in each individual region. Data analysis was performed with the peak-fit software of the spectrometer (Thermo Scientific Avantage Software, Waltham, MA, USA) using Gaussian functions with 20% Lorentzian component, and binding energy values were adjusted to the C1s transition (284.6 eV). This process has an associated error of ± 0.2 eV [37].

### 2.3. Photocatalytic Activity Measurements

Details of the photocatalytic activity measurements in the two studied reactions are presented next. 

#### 2.3.1. Photocatalytic Decomposition of Acetic Acid 

Photocatalytic tests were performed in a cylindrical quartz reactor (Heraeus, type UV-RS-2, Hanau, Germany) with a medium pressure mercury vapor lamp (TQ-150, λ_max_ = 365 nm, Hanau, Germany) located in the center of the vessel and surrounded by a water-circulating jacket. Gas inlet and outlet are in the upper part of the reactor. One molar solution of acetic acid (0.35 L) and the photocatalyst (0.35 g) were introduced in the reactor. A stream of He (60 mL/min) was bubbled through the reactor for 2 h to purge lines and remove dissolved oxygen and, then, the cooling system and the UV lamp were turned on. The process was conducted for 12 h under continuous magnetic stirring. Some experiments were repeated twice to check reproducibility. A blank experiment (without catalyst) was also performed in the same conditions to measure the photolysis effect. Analysis of the outlet gas stream was performed by mass spectrometry (Balzers, Thermostar GSD 301 01, Asslar, Germany). Calibrated cylinders of 2000, 2000 and 500 ppmv in helium of CH_4_, CO_2_ and H_2_, respectively, were used to quantify the products. The photocatalytic activity was expressed as indicated in Equation (7).

(7) $$\mathrm{Production}\;\mathrm{of}\;X=\frac{\mathrm{mmol}\;X}{\mathrm{mol}\;\mathrm{HAc}}$$

where X is CH_4_, CO_2_ or H_2_.

#### 2.3.2. Photocatalytic Oxidation of Propene

The experimental system used to perform the activity tests was designed in our laboratory and consists of a quartz reactor (AFORA, Fisher Scientific, Hampton, NH, USA) and a 365 nm Philips UV-A lamp placed parallel to the quartz reactor at about 1 cm. The assembly quartz reactor lamp is surrounded by a cylinder covered by tinfoil. A scheme of this system is shown elsewhere [8]. 

The photocatalyst (0.11 g) was placed on a quartz wool plug inside the reactor and then, after purging with helium, a stream of 100 ppmv propene in air was fed to the reactor at 25 °C, being the outlet gas continuously analyzed by mass spectrometry (MS, Balzers, Thermostar GSD 301 01, Asslar, Germany). Once the propene concentration is stable (after about 3 h), the lamp is switched on and kept working until a stationary propene signal is achieved (usually 3 h). All the tests were carried out with 30 and 60 mL/min propene flow. A blank experiment (without catalyst) was also performed in the same conditions to measure the photolysis effect. The experiments were repeated at least twice to check reproducibility. Propene conversion was calculated using Equation (8):

(8) $$\mathrm{Propene}\;\mathrm{conversion}\;\left(\%\right)=\frac{C_{\mathrm{initial}}{}_{C_{3}H_{6}}-C_{{\mathrm{stationary}}_{C_{3}H_{6}}}}{C_{\mathrm{initial}}{}_{C_{3}H_{6}}}\times 100$$

where C_initialC3H6_ is the initial propene concentration, 100 ppmv, and C_stationaryC3H6_ is the stationary propene concentration reached after a certain irradiation time. 

The produced CO_2_ was quantified using a calibrated cylinder (300 ppmv CO_2_ in helium). Water evolution was also followed by MS and a mass scan was carried out to determine if additional oxidation compounds were present in the outlet stream. An example of the type of information obtained in this type of experiment has been shown in a previous publication [38].

## 3. Results

### 3.1. Textural and Morphological Properties

**Figure 1:**
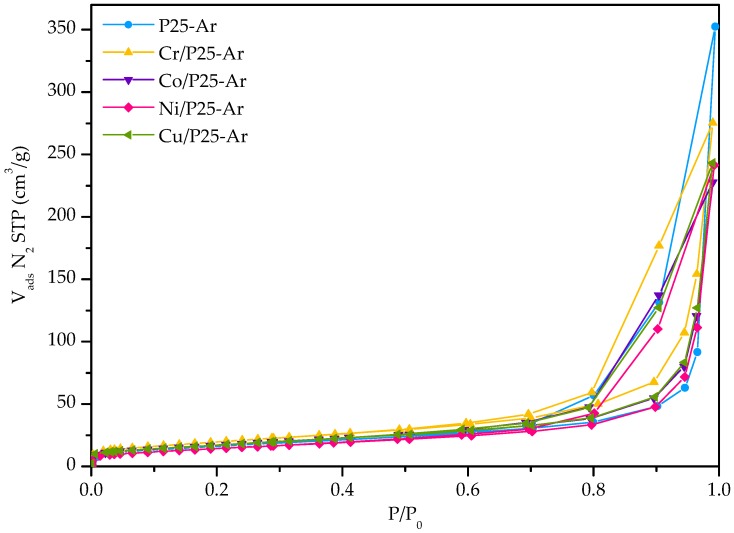
N_2_ adsorption–desorption isotherms at −196 °C of the photocatalysts.

Figure 1 shows the N_2_ adsorption–desorption isotherms of M/P25-Ar and P25-Ar samples. It can be observed that the adsorption isotherms are type IV, according to the IUPAC classification [39], with a hysteresis cycle typical of mesoporous materials. The incorporation of metallic species results in a slightly lower adsorption capacity than P25-Ar.

The parameters calculated from the adsorption isotherms (Figure 1) are collected in Table 1. It can be observed that all the M/P25 samples have similar textural properties, with mesopore volume, total pore volume and average pore diameter (Ø) slightly lower than those of P25-Ar. It can be mentioned that the surface area and micropore volume of Cr/P25-Ar are slightly higher than for the rest of samples. 

SEM analysis did not reveal noticeable morphological differences neither between the M/P25-Ar samples and P25-Ar, nor among the M/P25-Ar samples. Figure S1 (in Supplementary Information) shows the SEM images of P25-Ar and Cu/P25-Ar samples as examples. The SEM-EDX mapping image of Cu/P25-Ar shows the uniform distribution of the Cu species on TiO_2_ (see Figure S2).

### 3.2. X-ray Diffraction

**Figure 2:**
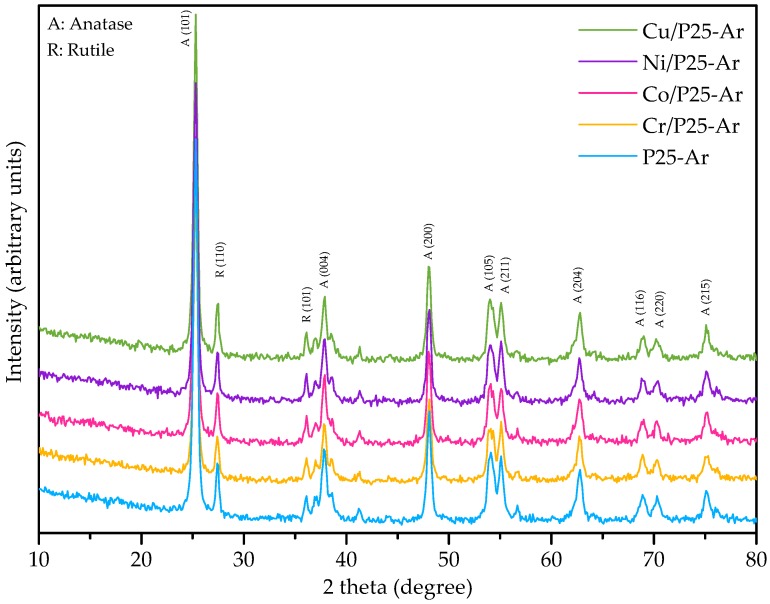
X-ray diffraction patterns of the photocatalysts.

Figure 2 shows the XRD patterns obtained for P25-Ar and M/P25-Ar samples. All of them present the characteristic peaks of anatase (2θ values of 25.3° (101), 37.8° (004), 48.0° (200), 54.5° (105), 55.0° (211), 62.7° (204), 70.4° (116), and 75.2° (220)) and rutile (2θ values of 27.5° (110), 36.1° (101) and 54.4° (211)) [40]. No characteristic peaks of transition metal oxides have been identified in the M/P25-Ar XRD patterns. This can be explained by the low metal loading, because the metal oxide particles are highly dispersed, and/or because the metallic ions have been partially introduced into the TiO_2_ structure.

Table 2 summarizes the average crystallite size of anatase and rutile and, also, the contribution of crystalline and amorphous TiO_2_ in each photocatalyst.

From data in Table 2 it can be observed that the addition of transition metallic species does not modify in a relevant way the TiO_2_ crystalline structure, the phases distribution or the crystal size of any of the phases. It can be mentioned that the Cr/P25-Ar sample presents the smallest rutile average crystal size, and this could be related to its slightly higher specific surface area (see Table 1).

### 3.3. UV-Vis Diffuse Reflectance Spectroscopy

**Figure 3:**
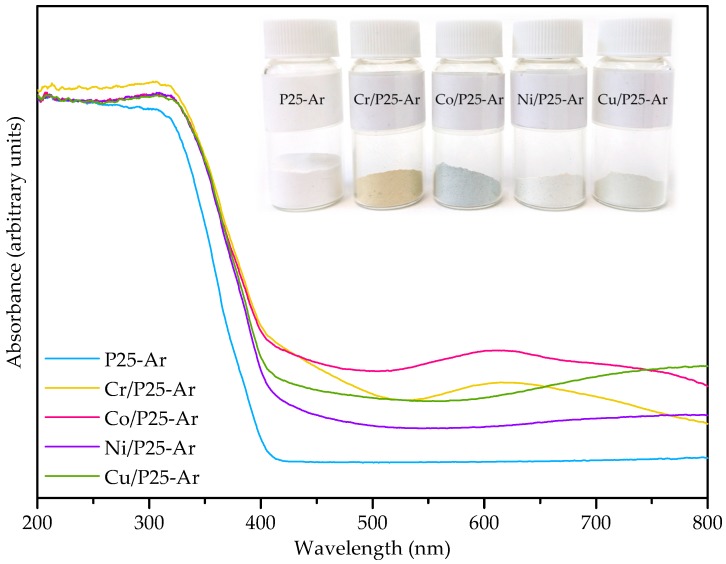
UV-vis diffuse reflectance spectra for bare P25-Ar and for M/P25-Ar (M = Cr, Co, Ni and Cu) catalysts. Inset: photograph of the prepared photocatalysts.

Figure 3 compiles the UV-vis diffuse reflectance spectra (DRS) of M/P25-Ar and P25-Ar samples. It can be observed that bare P25-Ar has low absorption in the visible region (>400 nm), while the M/P25 samples show higher absorption in this region. Moreover, the absorption edge for the M/P25-Ar samples is shifted to the visible range (423–436 nm), which results in a decrease in their band gap energy values. The absorption edge of the studied photocatalysts increases as follows: P25-Ar < Ni/P25-Ar < Cu/P25-Ar < Cr/P25-Ar < Co/P25-Ar (see Table 3). The inset in Figure 3 presents a photograph of the prepared samples, showing that they are coloured, which reveals the presence of metal oxides. 

The band gap (E_g_) values of the photocatalysts have been calculated by the absorbance method, considering the direct allowed transitions (direct method) and the indirect allowed transitions (indirect method); they are presented in Table 3. The data obtained by the different methods show a similar trend, being those calculated by the absorbance and indirect methods quite close, and lower than those calculated by the direct method. Furthermore, it can be observed that the difference in the E_g_ values of samples M/P25-Ar are not significant, and lower than that of P25-Ar.

As reported in the literature [19,20,27], the presence of transition metallic species, even in samples prepared by impregnation, might introduce new intra band gap states in the TiO_2_ structure, which could enhance their photoefficiency in the visible region. This behaviour can be mainly attributed to two phenomena: (i) these new energy levels would be located below the lower conduction band edge of TiO_2_, decreasing the TiO_2_ band gap and shifting the absorption band to the visible part of the spectrum; and (ii) these energy levels would also act as e^−^ traps, increasing the h^+^ lifetime and decreasing the e^−^/h^+^ recombination rate [14].

### 3.4. XPS

**Figure 4:**
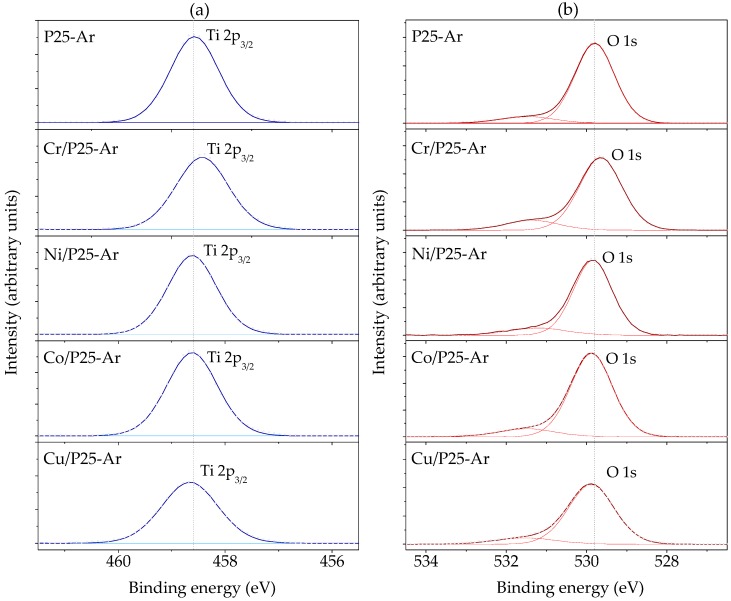
Deconvoluted XPS spectra for: (**a**) Ti 2p_3/2_ and (**b**) O 1s in P25-Ar and in the M/P25-Ar photocatalysts.

P25-Ar and the M/P25-Ar catalysts were analysed by XPS in order to study their surface composition and the oxidation state of the metal cations. Figure 4a,b show, respectively, the obtained Ti 2p_3/2_ and O 1s XPS spectra. The binding energy of Ti 2p_3/2_, about 458.6 eV (Figure 4a), indicates the presence of titanium in an octahedral anatase network [41], which is consistent with Ti (IV). In the case of the Cr/P25-Ar sample, the B.E. is slightly lower than in the rest of the samples, which according to the literature can be attributted to the presence of some Ti (III) on the surface [42].

The O 1s spectra (Figure 4b) show two contributions, one at about 529.8 eV, ascribed to the lattice O^−2^ in crystalline TiO_2_ [43], and a second one at 531.5 eV, that can be due to O^−^ ions located in defect sites related to grain boundaries [43]. It can be observed that the intensity of the peak at 531.5 eV is slighlty higher in Cr/P25-Ar, which is in agreement with the larger amount of oxygen vacancies proposed before. The differences between the Cr/P25-Ar sample and the rest of M/P25-Ar catalysts revealed by XPS are consistent with the previoulsy commented XRD and BET results. There is not a signal due to oxygen bonded to metallic species, which should appear at about 530 eV [41], probably due to the low metallic content present in the catalysts.

**Figure 5:**
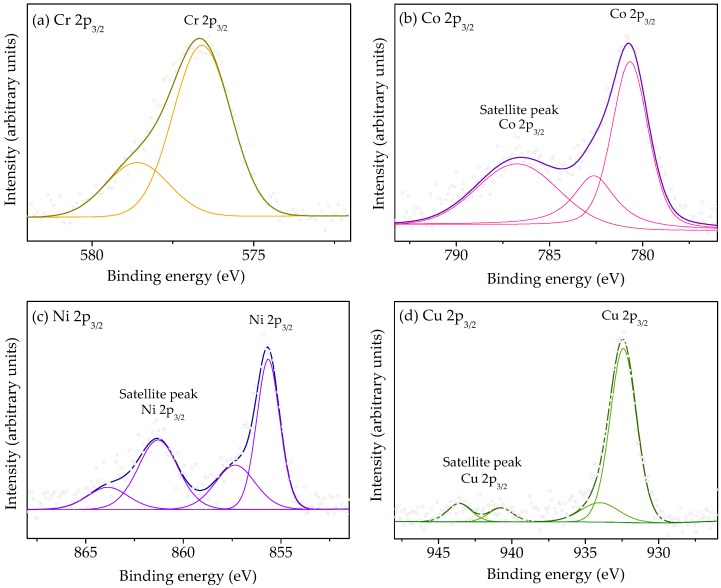
XPS deconvoluted spectra of: (**a**) Cr 2p_3/2_ in Cr/P25-Ar, (**b**) Co 2p_3/2_ in Co/P25-Ar, (**c**) Ni 2p_3/2_ in Ni/P25-Ar and (**d**) Cu 2p_3/2_ in Cu/P25-Ar.

Figure 5 shows the Cr 2p_3/2_, Co 2p_3/2_, Ni 2p_3/2_ and Cu 2p_3/2_ deconvoluted spectra obtained. 

The XPS spectra of Cr 2p_3/2_ in Cr/P25-Ar (Figure 5a) can be deconvoluted into two contributions: at 576.6 eV, assigned to Cr (III) species, and at 578.6 eV, which is attributed to Cr (VI) [44]. The Co 2p_3/2_ XPS spectra (Figure 5b) shows a main asymmetric peak located at 780.7 eV and a satellite peak positioned at 786.5 eV. These patterns reveal that Co/P25-Ar sample only contains Co (II) [37]. The Ni 2p_3/2_ spectra (Figure 5c) shows a main peak at 855.6 eV, ascribed to Ni (II) in an oxygen environment [45], a minor peak at 857.3 eV that corresponds to Ni (III) species [46], and a satellite peak, at 861.3 eV, that supports the presence of divalent nickel [45]. Finally, the Cu 2p_3/2_ XPS spectrum (Figure 5d) shows two contributions at 932.4 eV and 934.0 eV, that can be assigned, respectively, to Cu (I) and Cu (II) [43,47]. The Cu (II) state also leads to the shake-up satellite peaks at 940.8 and 943.6 eV [47]. 

Table 4 shows the binding energy values and the identified oxidation states for the metallic species, with the estimated proportions, in each sample. It can be mentioned that, in some cases, the metallic species show oxidation states different from those of the metallic ions in the salts used for impregnation, which can be explained by the interaction of the metallic precursor with TiO_2_, being copper species the most affected by this interaction. 

The metal content on the catalyst’s surface has been calculated from XPS data and a value around 3 wt.% was obtained for all the M/P25-Ar samples. Considering that the nominal (and very likely actual) metal loading is 1 wt.%, this reveals that the samples show a certain surface enrichment in metallic species on the more external surface of the catalyst.

### 3.5. Photocatalytic Activity

The results obtained in the study of the acetic acid photodecomposition in the liquid phase and the photooxidation of propene at low concentration in the gas phase are presented and discussed next.

#### 3.5.1. Photocatalytic Decomposition of Acetic Acid

**Figure 6:**
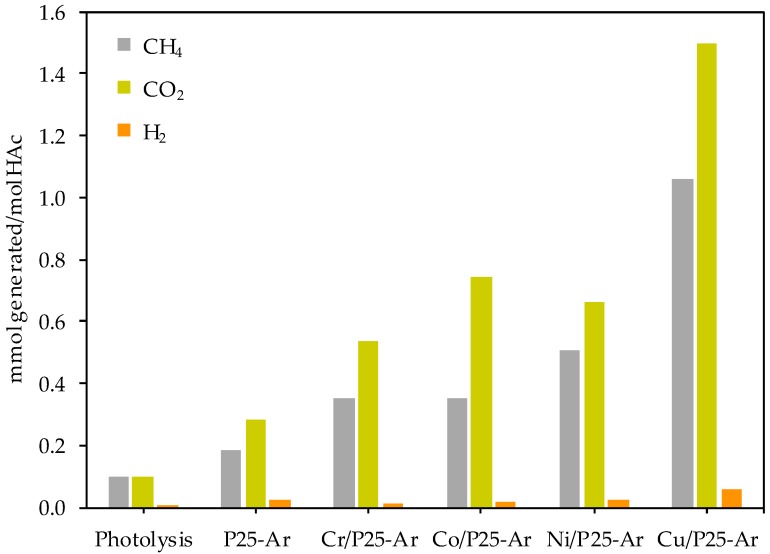
CH_4_, CO_2_ and H_2_ produced after 12 h with the prepared photocatalysts and in the absence of a photocatalyst.

Figure 6 plots the amount of CH_4_, CO_2_ and H_2_ (in mmol per mol of acetic acid (HAc)) generated during 12 h in the different catalytic tests performed. Because the H_2_ production is very low, the comments on the obtained results will be based on the production of CH_4_ and CO_2_. It can be observed that the produced amounts of CH_4_ and CO_2_ by photolysis are small compared to those produced in the presence of the photocatalysts.

Methane formation during the photocatalytic decomposition of HAc is supposed to follow the so-called photo-Kolbe reaction (Equation (9)),

CH_3_COOH + h^+^ → CH_3_^−^ + CO_2_ + H^+^ (9)

the main products of which, CH_4_ and CO_2_ [7,48,49,50], are ideally produced in CH_4_/CO_2_ molar ratio equal to 1. However, it is interesting to note that in the obtained results, the amount of CO_2_ produced is higher than the amount of CH_4_, being the CH_4_/CO_2_ ratio lower than one for all the tested catalysts. This means that some other reactions, besides the photo-Kolbe one, have to be taken into consideration. Mozia, et al. [48] suggested that the excess of CO_2_ could be originated by the oxidation of acetic acid with photogenerated O_2_, as shown in Equation (10):

CH_3_COOH + 2 O_2_ → 2 CO_2_ + 2 H_2_O (10)

Thus, it could be assumed that both the photo-Kolbe (Equation (9)) and the oxidation reaction (Equation (10)) occur simultaneously in the experiments carried out. If both reactions would occur up to the same extent, the theorethical CH_4_/CO_2_ ratio would be 0.30. However, the measured CH_4_/CO_2_ ratio ranges between 0.47 and 0.77, meaning that with the tested catalysts and conditions, the photo-Kolbe reaction is predominant, prevaling acetic acid degradation over its oxidation.

Figure 6 shows that M/P25-Ar photocatalysts produce more CH_4_ and CO_2_ than P25-Ar, confirming a clearly positive effect of the metal. Data of Figure 6 also indicate that the photocatalytic activity is significantly influenced by the nature of the added metal. Concerning the methane output, the photocatalytic efficiency of the M/P25-Ar samples follows the trend: Cu/P25-Ar >> Ni/P25-Ar > Cr/P25-Ar ≈ Co/P25-Ar. The acetic acid conversion varies between 0.01% and 0.13% for all of them, which corresponds, respectively, to 0.10 and 1.28 mmol of acetic acid converted per gram of photocatalyst in 12 h.

#### 3.5.2. Propene Oxidation

**Figure 7:**
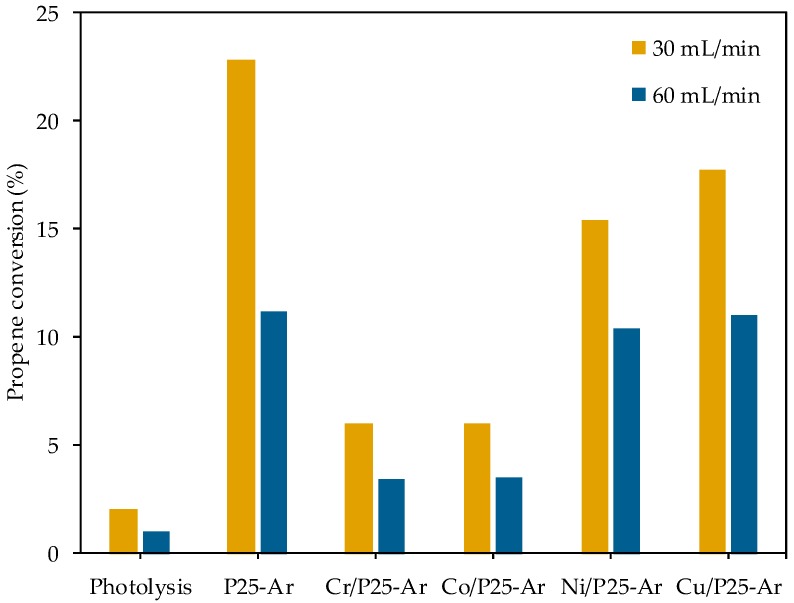
Propene conversion (at 30 and 60 mL/min) obtained without catalyst, and with P25-Ar and M/P25-Ar photocatalysts.

Figure 7 shows the propene conversion values obtained with the five prepared photocatalysts and in the blank experiment, tested at 30 and 60 mL/min. The mass scan carried out to determine oxidation compounds in the outlet stream reveals that CO_2_ is the only oxidation product. Also, quantification of the produced CO_2_ has allowed to perform a carbon balance which confirms that total mineralization of propene takes place, according to the following reaction (Equation (11)), and in agreement with the literature [8,51].

2 C_3_H_6_ + 9 O_2_ → 6 CO_2_ + 6 H_2_O (11)

Photodegradation of propene by photolysis is negligible compared with the photocatalytic oxidation. The activity order of the investigated photocatalysts follows the same trend in both flow-rates tested (P25-Ar > Cu/P25-Ar > Ni/P25-Ar > Cr/P25-Ar ≈ Co/P25-Ar), which can be considered as a proof of reproducibility. As expected, propene conversion is higher when the used flow is 30 mL/min. 

## 4. Discussion

As mentioned above, the photocatalytic activity of the M/P25-Ar samples in the two studied reactions varies with M, and the samples can be ordered according to their activity following a similar trend in both reaction media: Cu/P25-Ar > Ni/P25-Ar > Cr/P25-Ar ≈ Co/P25-Ar. Because of the similarity in the textural properties (Table 1), crystallinity and TiO_2_ phase distribution (Table 2) of the investigated photocatalysts, it can be concluded that the differences in activity found are not related with the mentioned properties. Thus, it must be considered that in both reactions the photocatalytic activity depends on the nature of the incorporated metallic species. As the metals have different electrochemical properties, their ability to influence the photocatalytic behaviour of titania can be different. The ionic radius and the work function are relevant parameters that would determine their behaviour. Table 5 lists the ionic radii [52] and the work functions [53] of each M species identified by XPS in the M/P25-Ar samples. 

The ionic radius of Cr (III) is similar to that of Ti (IV), and this would let Cr (III) replace Ti (IV) in the TiO_2_ lattice. Such a substitution in M/TiO_2_ samples, has been previously reported in samples also prepared by impregnation and heat-treated at about 500 °C, [27]. Moreover, the ionic radius of Cr (VI) is smaller than that of Ti (IV), so Cr (VI) species could also be incorporated in the TiO_2_ structure. The incorporation of Cr species in the TiO_2_ lattice might introduce some defects and oxygen vacancies (that could explain the slightly different properties of Cr/P25-Ar catalyst, revealed by N_2_ adsorption, XRD and XPS). All the other metals must be located either in the network interstices or as aggregates on the TiO_2_ surface. The latter is the most plausible option since no significant changes in the crystalline structure of these M/P25-Ar samples compared to P25-Ar have been observed. 

As indicated in the introduction, electron transfer can occur from lower work function materials to higher work function ones. In the case of the M/P25-Ar samples, the metal work function must be higher than that of TiO_2_ to achieve an efficient photocatalytic behaviour. The metallic species can, thus, behave as efficient traps for photogenerated electrons, preventing the e^−^/h^+^ recombination and, consequently, improving the photocatalytic activity [54].

The activity results obtained show that Cr/P25-Ar and Co/P25-Ar are the samples with the lowest photocatalytic activity in both reactions. In the case of the Co catalyst, it can be due to the lower work function of Co (II) ions with respect to that of Ti (IV). This would not allow the Co (II) ions to capture electrons in an efficient way [19,22,53]. As the Cr species present in Cr/P25-Ar have a high work function, the low activity must be related to a different property. As commented before, it seems that the Cr (III) ions partially substitute Ti (IV) in Cr/P25-Ar and probably because of that, the catalyst loses activity. In contrast, Ni/P25-Ar and Cu/P25-Ar show the highest performances in both reactions. This can be explained considering that these samples contain, respectively, Ni (II) and Cu (II) ions, that have higher work function than Ti (IV). Another advantage of copper is that, as reported in the literature [2,7,55,56], the conduction band (CB) edges of Cu_2_O and CuO species are less negative than that of TiO_2_, while the valence band (VB) edges of Cu_2_O and CuO are less positive than that of TiO_2_. This implies that in the Cu-P25-Ar sample, Cu_2_O and CuO introduce energy levels close to both the CB and VB of TiO_2_, which can act as effective e^−^ and h^+^ traps. Besides, the coexistence of the two Cu species generates electron transfer in cascade, from more positive energy values to less positive ones. This way of electron transfer may result in a better e^−^/h^+^ separation, associated to higher photoactivity.

In the photodecomposition of acetic acid, Cu/P25-Ar is clearly more active than the rest of M/P25-Ar photocatalysts. Besides, as mentioned above, all the M/P25-Ar samples are more active than P25-Ar. On the other hand, in the gas phase oxidation of propene, Ni/P25-Ar and Cu/P25-Ar show a close behavior, being bare P25-Ar more active than the M/P25-Ar photocatalysts. This indicates that the course of the two reactions seems to depend differently on the catalysts properties. Previous works compared the photoactivity of TiO_2_ systems in abatement of pollutants in liquid and gas phase and found that the photocatalysts’ behaviour was different in the two phases [57,58]. This can be related with either the own reaction mechanism, or to differences in experimental conditions, like the reaction media (liquid or gas phase), the reactor configuration or the substrate concentration. For example, as indicated in the literature [59,60,61,62], in the liquid phase, the solvent (usually water) helps to remove reaction intermediates and products from the catalyst surface, avoiding or hindering deactivation, whereas in fixed-bed reactors like the one used for propene oxidation, the light distribution becomes a limiting factor [63]. Probably, the gas phase photooxidation of propene is a rapid process occurring mainly on the titania surface. It seems that when the metallic species are present, the reaction rate decreases (i.e. by decrease of TiO_2_ exposed surface) and this is also influenced, as mentioned above, by the metal’s nature. In the liquid phase reaction, the diffusion limitations likely make the whole reaction slower and, thus, the pathway in which the supported metallic species are involved is not limiting the reaction. In this case, the positive effect of the metallic species as electron traps prevails. 

## 5. Conclusions

In the present work, a series of M/TiO_2_ photocatalysts (M = Cr, Co, Ni, Cu) were prepared by impregnation of the commercial P25 titania followed by heat treatment in Ar at 500 °C. 

The characterization results have shown that the textural properties of the M/P25-Ar photocatalysts are very close to those of bare P25-Ar, and only sample Cr/P25-Ar shows a slightly higher surface area. The crystalline structure of P25-Ar is, as well, almost not affected by the metal incorporation, and XRD patterns due to metal oxides have not been detected, which allows us to conclude that the metallic species are highly dispersed on the TiO_2_ surface. The crystal size of anatase and rutile was very similar in all M/P25-Ar samples, although in Cr/P25-Ar the average crystal size of rutile was the lowest. The band gap energies of the M/P25-Ar samples were a bit narrower than that for bare P25-Ar. The XPS analysis indicated that excepting Co, the rest of the metals are present in more than one oxidation state. The Ti 2p and O 1s spectra of Cr/P25-Ar show, compared to the rest of samples, a shift to lower binding energies, which suggests that Cr could have partially been incorporated in the TiO_2_ structure in this sample, leading to some defects. This is possible because the atomic radii of Cr (III) and Cr (VI) ions are smaller than that of Ti (IV), and it could explain the differences in the textural properties and crystal size of Cr/P25 compared to the rest of M/P25 photocatalysts. 

The photoactivity of the M/P25-Ar photocatalysts follows the same trend in the two studied reactions: Cu/P25-Ar > Ni/P25-Ar > Cr/P25-Ar ≈ Co/P25-Ar. To explain the differences between catalysts, the work functions and the ionic radii of the incorporated metallic ions have been considered to have a role. It has been concluded that the low activity of Cr/P25-Ar sample is related with the partial incorporation of Cr species in the TiO_2_ lattice, which results in lattice defects and oxygen vacancies that would facilitate recombination of electrons and holes. The low activity of Co/P25-Ar has been explained by the low work function of Co (II) compared to that of Ti (IV). The Cu/P25-Ar photocatalyst showed the best photocatalytic performance among the M/P25-Ar samples because Cu ions dispersed on TiO_2_, present as Cu (II) and Cu (I), have suitable energy levels of CB and VB edge to trap e^−^ and h^+^ and carry out an effective electronic transfer. 

Although the relative activity order of the metal-containing photocatalysts is the same in both reactions, P25-Ar shows better activity than Cu/P25-Ar in propene oxidation, whereas Cu/P25-Ar is more active than P25-Ar for acetic acid degradation. This can be explained considering that the rate-determining steps are significantly different in gas and liquid media. In the gas phase, propene oxidation is a rapid process that happens mainly on the TiO_2_ surface, and the supported metallic species reduce the exposed titania surface, decreasing the photocatalytic efficiency. In contrast, in the liquid phase, as the diffusion processes make the reaction slower, the positive effect of the metallic species as electron traps can be better identified.

## Figures and Tables

**Table 1:** Textural properties of the prepared materials.

Photocatalyst	S_BET_ (m^2^/g)	V_N2_ (cm^3^/g)	V_meso_ (cm^3^/g)	V_T_ (cm^3^/g)	Ø ^1^ (nm)
P25-Ar	58	0.02	0.48	0.53	36
Cr/P25-Ar	73	0.03	0.37	0.43	23
Co/P25-Ar	62	0.02	0.31	0.35	23
Ni/P25-Ar	53	0.02	0.34	0.37	28
Cu/P25-Ar	63	0.02	0.33	0.38	24

^1^ Average pore diameter.

**Table 2:** Crystalline properties determined from XRD patterns.

Photocatalyst	Average Crystal Size (nm)		Crystalline TiO_2_ (wt.%)		Amorphous TiO_2_ (%)
	Anatase	Rutile	Anatase	Rutile	
P25-Ar	19	32	77	11	12
Cr/P25-Ar	19	25	76	13	11
Co/P25-Ar	17	30	74	14	12
Ni/P25-Ar	20	32	76	12	12
Cu/P25-Ar	19	31	76	12	12

**Table 3:** Absorption edge wavelength and calculated E_g_ values for P25-Ar and M/P25-Ar photocatalysts.

Photocatalysts	Absorption Edge Wavelength (nm)	E_g_^1^ (eV)	E_g_^2^ (eV)	E_g_^3^ (eV)
P25-Ar	403	3.08	3.53	3.11
Cr/P25-Ar	436	2.85	3.40	2.86
Co/P25-Ar	436	2.84	3.40	2.88
Ni/P25-Ar	422	2.93	3.41	2.95
Cu/P25-Ar	426	2.91	3.39	2.92

^1^ Absorbance method. ^2^ Direct allowed transitions (direct method). ^3^ Indirect allowed transitions (indirect method).

**Table 4:** Binding energies and predominant oxidation states of the metal transition ions, determined from XPS spectra.

Photocatalyst	Binding Energy (eV)			Indentified Metal Oxidation States	Proportion ^1^ (%)
	Ti 2p_3/2_	O 1s	M 2p_3/2_		
P25-Ar	458.6	529.8	-	-	-
Cr/P25-Ar	458.4	529.7	576.6	Cr (III)	74
			578.6	Cr (VI)	26
Co/P25-Ar	458.6	529.9	780.7	Co (II)	100
Ni/P25-Ar	458.6	529.8	855.7	Ni (II)	66
			857.3	Ni (III)	34
Cu/P25-Ar	458.7	529.9	932.4	Cu (I)	86
			934.1	Cu (II)	14

^1^ Data obtained from the characteristic XPS peak areas for each oxidation state of the metallic species.

**Table 5:** Ionic radii and work function values for the predominant oxidation states of the transition metal ions in the photocatalysts.

Identified Metal Oxidation States ^1^	Ionic Radius ^2^ (Å)	Work Function ^3^ (eV)
Ti (IV)	0.75	5.4 ± 0.2
Cr (III)	0.76	5.0 ± 0.2
Cr (VI)	0.58	6.8 ± 0.2
Co (II)	0.88	4.6 ± 0.2
Ni (II)	0.83	6.3 ± 0.2
Ni (III)	0.74	4.9 ± 0.1
Cu (I)	0.91	4.9 ± 0.1
Cu (II)	0.87	5.9 ± 0.1

^1^ Data obtained from XPS. ^2^ Obtained from reference [52]. ^3^ Obtained from reference [53].

## Supplementary Materials

The following are available online at http://www.mdpi.com/1996-1944/12/1/40/s1, Figure S1: scanning electron microscope (SEM) images of (**a**,**b**) P25-Ar and (**c**,**d**) Cu/P25-Ar samples with: 10 μm scale bar (**a**,**c**) and 1 μm scale bar (**b**,**d**). Figure S2: Scanning electron microscopy with energy dispersive X-ray (SEM-EDX) mapping for Cu/P25-Ar sample: Ti (red) and Cu (green).
