# Supplementary material for: TiO2 Modification with Transition Metallic Species (Cr, Co, Ni, and Cu) for Photocatalytic Abatement of Acetic Acid in Liquid Phase and Propene in Gas Phase
Source: Materials (Basel). 2018 Dec 23;12(1):40. doi: 10.3390/ma12010040 (PMC6337716; doi:10.3390/ma12010040)
Supplement: Supplementary file 1 [file materials-12-00040-s001.pdf]

materials

Supplementary Materials: TiO_2 Modification with Transition Metallic Species (Cr, Co, Ni, and Cu) for Photocatalytic Abatement of Acetic Acid in Liquid Phase and Propene in Gas Phase

Ana Amorós-Pérez, Laura Cano-Casanova, Ana Castillo-Deltell, María Ángeles Lillo-Ródenas * and María del Carmen Román-Martínez

MCMA Group, Department of Inorganic Chemistry and Materials Institute, University of Alicante, E-03080 Alicante, Spain; ana.amoros@ua.es (A.A.-P.); laura.cano@ua.es (L.C.-C.); ana.castillo@hotmail.es (A.C.-D); mcroman@ua.es (M.C.R.-M.).

* Correspondence: mlillo@ua.es; Tel.: +34965903545; Fax: +34965903454

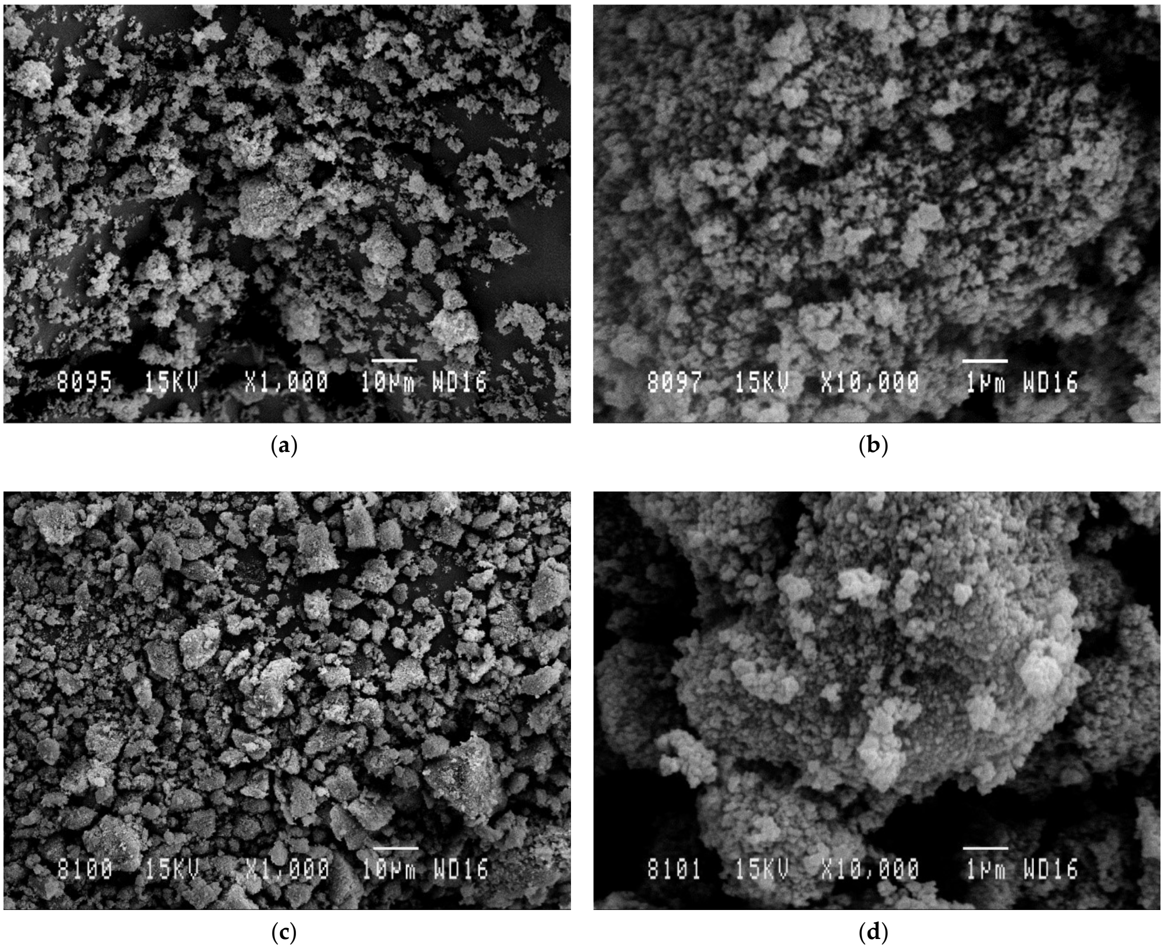

Figure S1. scanning electron microscope (SEM) images of (**a**,**b**) P25-Ar and (**c**,**d**) Cu/P25-Ar samples with: 10 µm scale bar (**a**,**c**) and 1 µm scale bar (**b**,**d**).

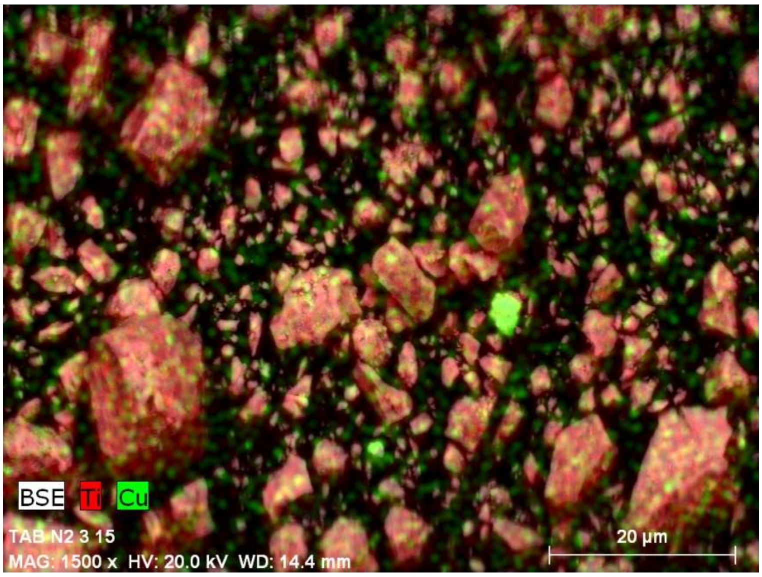

Figure S2. Scanning electron microscopy with energy dispersive X-ray (SEM-EDX) mapping for Cu/P25-Ar sample: Ti (red) and Cu (green).
